# Comorbidity, Criminality, and Costs of Patients Treated for Gambling Disorder in Denmark

**DOI:** 10.1007/s10899-023-10255-6

**Published:** 2023-10-09

**Authors:** Søren Viborg Vestergaard, Sinna Pilgaard Ulrichsen, Christian Møller Dahl, Thomas Marcussen, Christian Fynbo Christiansen

**Affiliations:** 1 Department of Clinical Epidemiology, Aarhus University Hospital, Olof Palmes Allé 43-45, 8200 Aarhus N, Denmark; 2 Department of Clinical Medicine, Aarhus University, Aarhus, Denmark; 3 Department of Clinical Pharmacology, Aarhus University Hospital, Aarhus, Denmark; 4 Department of Economics, University of Southern Denmark, Odense, Denmark; 5 Department of Economics and Business Economics, Aarhus University, Aarhus, Denmark; 6 Research Clinic on Gambling Disorders, Aarhus University Hospital, Aarhus, Denmark

**Keywords:** Gambling, Comorbidity, Criminal behavior, Drug therapy, Costs and cost analysis, Epidemiology

## Abstract

Gambling disorder is associated with increased mental comorbidity, unhealthy lifestyle, criminality, and costs-of-illness, but the available evidence is mainly based on self-reported survey data. We examined the registry-recorded mental and somatic comorbidities, medication use, criminality, and costs-of-illness associated with gambling disorder. We identified individuals diagnosed with or treated for gambling disorder in hospitals or specialized treatment centers during 2013–2017 and matched them by age and sex to general population comparisons. Using individual-level healthcare and socioeconomic registries, we characterized their history of mental and somatic comorbidities, medication use, and criminality. We estimated their cost-of-illness of welfare services (direct) and lowered productivity (indirect) using the human capital approach. We identified 1381 individuals with gambling disorder, primarily young (median age: 34 years) men (87%). Individuals with gambling disorder more frequently than their comparisons had previous hospital-recorded comorbidity [e.g., myocardial infarction (0.8% vs. 0.5%)], medication use [e.g., respiratory system drugs (35.6% vs. 28.6%)], and hospital-recorded or pharmacologically treated mental comorbidity [e.g., depression (39.8% vs. 14.9%)]. Also, sentenced criminality was much more common in individuals with gambling disorder (7.0%) than in comparisons (1.1%). The estimated attributable direct costs were €4.0 M corresponding to €2.9 K per person with gambling disorder, and attributable indirect costs were €17.6 M, corresponding to €13.2 K per person with gambling disorder in 2018. In conclusion, individuals diagnosed with or treated for gambling disorder have a high burden of mental and somatic comorbidities as well as criminality compared with the general population. This needs attention to minimize the societal and personal costs of gambling disorder.

**Supplementary Information:**

The online version contains supplementary material available at 10.1007/s10899-023-10255-6.

## Introduction

Gambling disorder affects millions of people worldwide with huge consequences on both individual and societal level (Calado & Griffiths, 2016). Over the past decades, the past-year prevalence of gambling disorder has been reported to be 0.02–3.4% in developed countries (Calado & Griffiths, 2016; Browne et al., 2017; Annalisa Belloni, 2021). Yet, only a fraction of those with gambling problems seek specialized treatment (Suurvali et al., 2008). Recently, a Danish population survey estimated that 0.7% of adults in Denmark in 2021 had a serious gambling problem (Rambøll, 2022). This number is three-fold higher than in 2016 and four-fold higher than in 2006 (Bonke, 2006; Fridberg, 2016). Similarly, a Norwegian survey reported an increase in prevalence of serious gambling problems from 0.9% in 2015 to 1.4% in 2019 (Kristensen et al., 2022). Therefore, gambling disorder and problems related to this may increase in the future.

Comorbid mental disease and unhealthy lifestyle are frequent in individuals with gambling disorder (Lorains et al., 2011). Comorbid mental disorders have been reported in 73% of those with gambling disorder (Håkansson et al., 2018), including depression and bipolar disorder (23–38%), alcohol abuse (17–28%), anxiety (18–37%), and drug abuse (7–17%) (Lorains et al., 2011; Dowling et al., 2015; Håkansson et al., 2018). Mental disorders commonly evolve prior to gambling problems, but can also develop as complications to gambling (Sundqvist & Rosendahl, 2019). Finally, unhealthy lifestyle (e.g., alcohol, smoking) is more common in individuals with gambling disorder (Dowling et al., 2015; Ford & Håkansson, 2020; Lorains et al., 2011). Yet, the risk of somatic lifestyle-related disease in individuals with gambling disorder remains unaddressed.

Moreover, criminal charges were more common among Danish individuals with a self-reported gambling problem (23%) than for individuals without (10%), while differences in age, sex, educational level, and use of alcohol and tobacco could not explain this association (Laursen et al., 2016). Correspondingly, an Australian survey found that 22% of individuals with self-reported problem gambling stated to have committed crimes during the previous twelve months (Browne et al., 2017). However, these surveys may be biased by selection of voluntary respondents, and there are no figures of registry-recorded criminality in individuals with diagnosed gambling disorder.

In recent studies, the total annual societal costs have been estimated at €1.42 billion in Sweden (Hofmarcher et al., 2020), and €5.14 billion corresponding to almost €9000 per individual with a gambling problem in Norway (Kristensen et al., 2022). Of the total costs, the major part was attributed to indirect (47–59%) and immaterial costs (28–37%), whereas direct costs accounted for less (13–16%) (Hofmarcher et al., 2020; Kristensen et al., 2022). These studies were only partly based on individual-level data (Hofmarcher et al., 2020; Kristensen et al., 2022), and additionally, the societal costs of gambling have been estimated in the UK and Australia based on extrapolation of average figures (Browne et al., 2017; Annalisa Belloni, 2021).

In summary, there is a need for studies of comorbidity, criminality, and costs of gambling disorder based on individual-level, non-self-reported data. Therefore, we aimed to estimate the occurrence of individuals seeking specialized treatment and describe their psychiatric and somatic comorbidities, criminal records, and cost of welfare services and lost productivity.

## Methods

### Study Design and Participants

#### Setting

This study was based on nationwide healthcare and socioeconomic databases in Denmark (~ 5.8M population) supplemented by data from treatment centers offering specialized treatment of gambling disorder. Danish healthcare is primarily tax-funded with equal access for all residents (Schmidt et al., 2019), including treatment for gambling disorder in specialized centers. Data were linked on individual level across databases using the unique personal identification number assigned to every person living in Denmark (Schmidt et al., 2019).

#### Gambling Disorder Cohort

We sampled a gambling disorder cohort from two data sources. First, we identified every person with a hospital-registered diagnosis of gambling disorder (F63.0 as primary or secondary diagnosis) in Denmark during 2013–2017 at discharge from inpatient or outpatient hospital courses according to the *International Classification of Diseases, Tenth Revision* (ICD-10) in the Danish National Patient Registry (Schmidt et al., 2015, 2019). Second, as gambling disorder is mainly treated outside hospitals, we also obtained data for the same period from centers offering specialized treatment to identify individuals treated for gambling disorder. During the inclusion period, such treatment was mainly offered by four major centers in Denmark: *Center for Ludomani*, *Mindwork*, *Behandlingscenter Tjele*, and *Research Clinic on Gambling Disorders at Aarhus University Hospital*. Each person could be included maximum once with date of inclusion defined as date of hospital diagnosis or date of first contact/initiated treatment registered in a treatment center, whichever occurred first. Of note, the extent of data from the treatment centers increased throughout the study period, reaching the expected level of completeness in the final years of the study period.

#### Newly Registered Gambling Disorder Cohort

Among the full gambling disorder cohort, we identified a cohort of all individuals with newly registered gambling disorder in 2017, i.e., individuals without any registered diagnosis/treatment during 1995–2016.

#### Comparison Cohort

For each individual in the gambling disorder cohort and newly registered gambling disorder cohort, we identified 1000 living individuals of the same sex and age in years from the general population to generate a comparison cohort (sampled with replacement) (Heide-Jørgensen et al., 2018; Schmidt et al., 2014). Individuals in the comparison cohort could not have a registered gambling disorder prior to the index date of the individual with a gambling disorder. The index date of matching was 1 January 2018 for the gambling disorder cohort, and the index date for the newly registered gambling disorder cohort was the date of the gambling disorder diagnosis or treatment initiation.

### Statistical Analyses

#### Occurrence

We estimated the occurrence (prevalence) of diagnosed or treated gambling disorder as number of individuals in the gambling disorder cohort alive on 1 January 2018 divided by number of adults in the total population. The occurrence of new cases (incidence) of gambling disorders per 100,000 person-years was computed as number of individuals in the newly registered gambling disorder cohort divided by the total person-years, i.e., the average population count in 2017.

#### Comorbidity, Criminality, and Use of Medical Drugs

We described the gambling disorder cohort and comparisons in terms of: age and sex (Schmidt et al., 2014), proportion with previous use of medical drugs (Pottegard et al., 2016), criminality (sentences), and comorbidity including the 19 chronic disorders from the Charlson Comorbidity Index (CCI) and 25 neurologic and psychiatric disorders (Charlson et al., 1987; Thygesen et al., 2011; Vestergaard et al., 2020).

#### Mortality

We followed individuals with newly registered gambling disorder and comparisons up to three years from inclusion or until the first of death or 31 December 2020 using data from the Danish Civil Registration System (Schmidt et al., 2014). We estimated the crude 3-year mortality rates per 1000 person-years at risk and compared the mortality of individuals with newly registered gambling disorder to comparisons with crude and adjusted hazard ratios (HRs) in a stratified Cox regression adjusted for differences in age, sex, and comorbidity.

#### Costs of Illness

We estimated the societal costs of gambling disorder using the human capital approach based on a model developed to estimate the costs of neurologic and mental disorders in Denmark (Vestergaard et al., 2020). We compared the direct and indirect costs of illness for every individual with a gambling disorder and their comparisons, estimating the annual costs both overall and per person. We computed both actual direct costs (i.e., cost of healthcare services) and attributable direct costs (i.e., the costs of individuals with gambling disorder minus the costs of comparisons divided by 1000 [to account for the 1:1000 matching ratio]).

Direct costs included individual-level data on all primary care, secondary care, and welfare services including general practitioners, practicing medical specialists, and dentist services from the Danish Health Service Registry (Andersen et al., 2011); medication expenditures on filled prescriptions from the Danish National Prescription Registry (Pottegard et al., 2017); nursing home or sheltered accommodation, personal nursing and other types of personal care, home nurse visits, and hospital-based neurorehabilitation from Statistics Denmark (Statistics Denmark); and hospital inpatient admissions, outpatient specialist clinic visits, and emergency room contacts and in-hospital medication from the Danish National Patient Registry (Ankjaer-Jensen et al., 2006).

The attributable indirect costs included the estimated loss of productivity defined as yearly income before taxes in individuals with gambling disorder subtracted from the yearly income in the comparison cohort.

For individuals with a newly registered gambling disorder, we estimated the costs during the first year from first recorded diagnosis or initiated treatment including the same cost components as above.

Costs were based on the current tariffs at the time of the services and adjusted according to the net price index (2015 = 100) from Statistics Denmark.

The analyses were performed using SAS version 9.4 (Cary, NC, USA). The study was registered at Aarhus University (record number 2016-051-000001/603) and approved by Statistics Denmark (project ID 706794) with legal permission from Central Denmark Region to transfer medical record data (case ID: 1-45-70-91-21) according to the Danish Health Care Act with a waiver for informed consent from the patients. No approval from an ethics committee was required for the study.

## Results

### Occurrence

We identified 1381 individuals diagnosed with or treated for gambling disorder alive as of 1 January 2018 corresponding to a 5-year prevalence of 0.02% (Suppl. Table 1). Almost one third (n = 411) was registered with both a hospital diagnosis and as treated in a specialized clinic, slightly more (n = 628) were identified solely as treated in a specialized clinic, and fewer (n = 342) were registered only with a hospital diagnosis of gambling disorder. We found 532 individuals with a first-time diagnosis/treatment course (newly registered gambling disorder) in 2017, corresponding to an average incidence of 9.2 per 100,000 person-years (Suppl. Table 1).

### Age and Sex Distribution

**Fig. 1:**
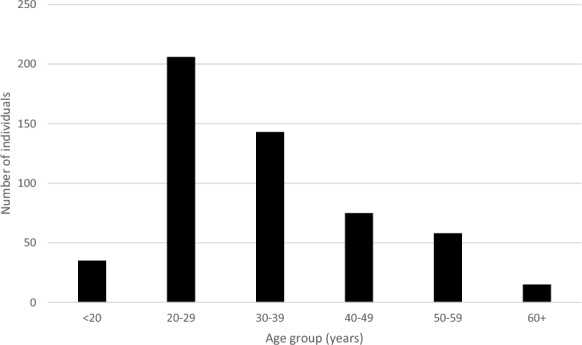
Age of individuals with newly registered gambling disorder at time of hospital diagnosis or initiated specialized treatment in Denmark during 2017

Most of the included individuals with gambling disorder were men (87%) and young adults (median age of 34 years, Table 1). In Fig. 1, we illustrated the age at the time of the first gambling disorder diagnosis.

**Table 1:** Age, sex, and comorbidity burden among individuals with gambling disorder and their matched comparisons from the general population

	Individuals with gambling disorder	Comparisons
No. of individuals, N (%)	1381 (100)	1,381,000 (100)
Age in years, median (25%;75% percentile)	34 (27;46)	34 (27;46)
Sex, N (%)	1203 (87.1)	1,203,000 (87.1)
Comorbidity burden, N (%)*		
No (CCI-score = 0)	1102 (79.8)	1,184,543 (85.8)
Mild-moderate (CCI-score = 1–2)	248 (18.0)	178,599 (12.9)
High (CCI-score = + 3)	31 (2.2)	17,858 (1.3)

*As defined in the Charlson Comorbidity Index (CCI)

### Comorbidity and Use of Medication

All hospital-recorded somatic comorbidities (CCI score > 0) were more common among pathological gamblers than comparisons both concerning mild-moderate scores (18% vs 13%) and high scores (2.2% vs 1.3%) (Table 1). Specifically, we observed a higher proportion of chronic pulmonary disease in individuals with gambling disorder (14.7%) than in comparisons (9.1%) (Table 2). Other types of comorbidity were less common, but still more common among individuals with gambling disorder than among the comparisons. Examples of this are myocardial infarction (0.8% vs 0.5%), peptic ulcer disease (0.7% vs 0.3%) and diabetes without end organ damage (2.5% vs 1.5%, Table 2).

**Table 2:** Somatic comorbidity among individuals with gambling disorder and their matched comparisons from the general population, including disorders from the Charlson Comorbidity Index

	Individuals with gambling disorder	Comparisons
No. of individuals, N (%)	1381 (100)	1,381,000 (100)
*Type of comorbidity during preceding 10 years*		
Myocardial infarction	11 (0.8)	6257 (0.5)
Congestive heart failure	5 (0.4)	4225 (0.3)
Peripheral vascular disease	8 (0.6)	5772 (0.4)
Cerebrovascular disease	15 (1.1)	13,274 (1.0)
Dementia	< 5	< 5
Chronic pulmonary disease	203 (14.7)	125,211 (9.1)
Connective tissue disease	8 (0.6)	9947 (0.7)
Peptic ulcer disease	9 (0.7)	4670 (0.3)
Mild liver disease	10 (0.7)	6954 (0.5)
Diabetes (type 1 or type 2)	35 (2.5)	20,204 (1.5)
Hemiplegia	< 5	< 5
Moderate to severe renal disease	9 (0.7)	6807 (0.5)
Diabetes with end organ damage (type 1 or type 2)	13 (0.9)	6725 (0.5)
Any tumor	19 (1.4)	18,587 (1.3)
Leukemia	< 5	< 5
Lymphoma	< 5	< 5
Moderate to severe liver disease	< 5	< 5
Metastatic solid tumor	< 5	< 5
AIDS	< 5	< 5

From Table 3, it appears that individuals with gambling disorder have often been previously diagnosed with other types of mental and neurologic disorders. When contrasted with the comparisons, individuals with gambling disorder were more often diagnosed with other addictions, e.g., alcohol abuse (11.9% vs 2.6%) and drug abuse (13.0% vs 2.2%); or with comorbid anxiety disorders (30.7% vs 11.1%); bipolar disorder (5.2% vs 0.5%); depression (39.8% vs 14.9%); developmental and behavioral disorders (9.6% vs 3.8%), personality disorders (10.6% vs 1.6%), schizophrenia spectrum disorders (10.2% vs 1.8%), or sleep disorders (17.3% vs 8.8%, Table 3).

**Table 3:** Comorbid neurologic and mental disorders among individuals with gambling disorder and their matched comparisons from the general population

	Individuals with gambling disorder	Comparisons
No. of individuals, N (%)	1381 (100)	1,381,000 (100)
*Disorder during preceding 10 years*		
Alcohol abuse	165 (11.9)	36,053 (2.6)
Anxiety disorders	424 (30.7)	153,294 (11.1)
Bipolar disorder	72 (5.2)	7241 (0.5)
Brain tumors	< 5	< 5
Cerebral palsy	5 (0.4)	3447 (0.2)
Dementia	< 5	< 5
Depression	550 (39.8)	206,044 (14.9)
Developmental and behavioral disorders	133 (9.6)	51,981 (3.8)
Drug abuse	180 (13.0)	30,468 (2.2)
Eating disorders	5 (0.4)	2031 (0.1)
Epilepsy	29 (2.1)	21,282 (1.5)
Headache	90 (6.5)	81,805 (5.9)
Infections of the central nervous system	14 (1.0)	8547 (0.6)
Intellectual disability	20 (1.4)	11,007 (0.8)
Multiple sclerosis	< 5	< 5
Neuromuscular disorders	< 5	< 5
Other neurodegenerative disorders	0 (0.0)	416 (0.0)
Parkinson’s disease	8 (0.6)	1684 (0.1)
Personality disorders	147 (10.6)	22,213 (1.6)
Polyneuropathy	7 (0.5)	4586 (0.3)
Schizophrenia spectrum disorders	141 (10.2)	25,380 (1.8)
Sleep disorders	239 (17.3)	122,072 (8.8)
Stress related disorders	325 (23.5)	65,513 (4.7)
Stroke	19 (1.4)	11,120 (0.8)
Traumatic brain injury	27 (2.0)	13,311 (1.0)

Correspondingly, individuals with gambling disorder had more commonly filled prescriptions in outpatient pharmacies up to five years before inclusion (Table 4). Contrasted with the comparisons, individuals with gambling disorder more commonly used medical drugs targeting the alimentary tract and metabolism (30.8% vs 22.8%), the nervous system (58.3% vs 37.8%), the respiratory system (35.6% vs 28.6%), and anti-infectives (70.2% vs 58.3%, Table 4).

**Table 4:** Use of medical drugs (filled prescriptions) among individuals with gambling disorder and their matched comparisons from the general population

	Individuals with gambling disorder	Comparisons
No. of individuals, N (%)	1381 (100)	1,381,000 (100)
*Prescriptions ATC group during preceding five years*		
Alimentary tract and metabolism	426 (30.8)	314,310 (22.8)
Blood and blood forming organs	104 (7.5)	79,815 (5.8)
Cardiovascular system	335 (24.3)	272,955 (19.8)
Dermatologicals	630 (45.6)	539,517 (39.1)
Genitourinary system and sex hormones	187 (13.5)	146,440 (10.6)
Systemic hormonal preparations, excl. Sex hormones and insulins	132 (9.6)	99,186 (7.2)
Anti-infectives for systemic use	969 (70.2)	804,436 (58.3)
Antineoplastic and immunomodulating agents	14 (1.0)	13,415 (1.0)
Musculo-skeletal system	611 (44.2)	490,655 (35.5)
Nervous system	805 (58.3)	522,028 (37.8)
Antiparasitic products, insecticides, and repellents	181 (13.1)	151,689 (11.0)
Respiratory system	491 (35.6)	395,615 (28.6)
Sensory organs	400 (29.0)	353,267 (25.6)
Various	5 (0.4)	8621 (0.6)

ATC, Anatomical therapeutic classification

### Criminality

We found that criminality was much more common in individuals with gambling disorder (7.0%) than in comparisons (1.1%, Table 5). Among individuals with gambling disorder, 3.5% had an unsuspended/partly suspended sentence, and 4.1% had a suspended sentence in the past five years (Table 5). Fraud (3.3%), burglary (2.1%), and common assault (1.9%) were the most common types of crimes committed by individuals with gambling disorder (Table 5).

**Table 5:** Criminal offences in the past five years among individuals with gambling disorder during 2013–2017 and their matched comparisons from the general population

	Individuals with gambling disorder	Comparisons
No. of individuals, N (%)	1381 (100)	1,381,000 (100)
*Type of sentence*		
Unsuspended/partly suspended	48 (3.5)	5663 (0.4)
Suspended	57 (4.1)	10,045 (0.7)
Fines, disqualification of driving license, etc	6 (0.4)	568 (0.0)
*Type of crime*		
Shoplifting	6 (0.4)	1039 (0.1)
Burglary	29 (2.1)	3417 (0.2)
Fraud	46 (3.3)	2770 (0.2)
Forgery	7 (0.5)	861 (0.1)
Common assault	26 (1.9)	8300 (0.6)
*At least one sentence of any type at any time prior to 2018*	97 (7.0)	15,325 (1.1)

### Mortality

Based on few deaths during the first three years after a newly registered gambling disorder, we observed a lowered mortality compared with the general population, though with estimates of large uncertainty (crude HR of 0.95 [95% CI 0.31–2.94]; adjusted HR 0.50 [95% CI 0.16–1.58], Table 6).

**Table 6:** Three-year mortality among individuals with newly registered gambling disorder during 2017 compared with comparisons from the general population matched by age and sex

	No. individuals, N	No. deaths, N	Risk time	Deaths per 1000 person-years	Crude hazard ratio (95% CI)	Adjusted hazard ratio (95% CI)*
Comparisons (general pop.)	532,000	3121	1,822,803	1.7	1.00 (ref)	1.00 (ref)
Newly registered gambling disorder	532	**	**	1.6	0.95 (0.31–2.94)	0.50 (0.16–1.58)

CI, Confidence interval

*Hazard ratio (HR) adjusted for sex, age, and comorbidity (somatic or psychiatric)

**Estimates not shown as they are based on fewer than five individuals (requirement by Danish law)

### Costs of Illness

The actual direct costs of healthcare services in individuals with gambling disorder were €5767 K in 2018, primarily due to costs of hospital admissions accounting for €3395 K. Of the total direct costs, we estimated that €4036 K were attributable to gambling disorder, corresponding to €2934 per person with gambling disorder in 2018 (Table 7).

**Table 7:** Direct and indirect costs during 2018 among individuals with registered gambling disorder in Denmark

	Individuals with gambling disorder			
	Attributable costs (thousand EUR)	Attributable cost per person (EUR)	Actual costs (thousand EUR)	Actual cost per person (EUR)
*Direct costs*				
Primary sector	88	64	354	257
Secondary sector—Outpatient	865	629	1420	1032
Secondary sector—Inpatient	2703	1965	3395	2468
Filled prescriptions	367	266	548	399
Home care	15	11	50	36
Nursing home/sheltered accommodation	*	*	*	*
Total direct costs	4036	2934	5767	4192
*Indirect costs*				
Lost productivity associated with illness	17,623	13,152	–	–
Lost productivity due to premature death	*	*		
Total indirect costs	*	*		

*Estimates not shown as they are based on fewer than five individuals (requirement by Danish law)

The attributable indirect costs of gambling disorders in 2018 were €17,623 K, corresponding to €13,152 per person with gambling disorder (Table 7). Of note, costs associated with premature death were not included due to the Danish privacy rules that do not allow reporting of data based on less than five individuals.

Hospital admissions also accounted for most of the attributable direct costs of healthcare services during the first year after inclusion with newly registered gambling disorder (Table 8). The attributable direct costs during the first year in individuals with newly registered gambling disorder in 2017 were €1268 K, corresponding to €2392 per person (Table 8). Finally, we estimated the average lost productivity per person with newly registered gambling disorder to be €7840 (Table 8).

**Table 8:** Attributable costs during first year after date of newly registered gambling disorder during 2017

	Newly registered gambling disorder 2017	
	Attributable costs (thousand EUR)	Attributable cost per person (EUR)
*Direct costs*		
Primary sector	30	56
Secondary sector—Outpatient	541	1021
Secondary sector—Inpatient	675	1272
Filled prescriptions	30	56
Home care	− 7	− 14
Nursing home/sheltered accommodation	*	*
Total direct costs	1268	2392
*Indirect costs*		
Lost productivity associated with illness	4022	7840
Lost productivity due to premature death	*	*
Total indirect costs	*	*

*Estimates not shown as they are based on fewer than five individuals (requirement by Danish law)

## Discussion

We established the largest gambling disorder cohort in Denmark to date including 1381 individuals and examined their comorbidities, medication use, criminality, mortality, and costs of illness compared with the general population. Using individual-level data, we showed that individuals with gambling disorder more commonly suffered from comorbid mental disorders such as addiction, mood disorders, and anxiety, but also somatic diseases such as chronic pulmonary diseases. We also found that individuals with gambling disorder more frequently used prescribed medication. Due to few deaths in the *newly registered gambling disorder cohort* during the study period, the mortality estimates lacked precision. Criminality in general, and specifically fraud, burglary, and common assaults were more common among those with gambling disorder. Finally, gambling disorder was associated with considerable attributable direct and indirect societal costs.

### Incomplete Data Coverage

Based on our clinical experience and findings from existing literature, we expected gambling disorder to be underreported in the hospital data, i.e., the data from the Danish National Patient Registry (Laursen et al., 2016; Rambøll, 2022; Suurvali et al., 2008). Therefore, we collected individual-level data files from every major specialized treatment center in Denmark to link these with individual-level data from existing registries. We want to emphasize that we analyzed the occurrence of diagnosed or treated gambling disorder, and not the total occurrence of gambling problems in the population.

### Somatic Comorbidity

Our finding of an association between gambling disorder and chronic pulmonary diseases is likely explained at least partly by the frequent use of tobacco in these patients (Dowling et al., 2015; Ford & Håkansson, 2020; Lorains et al., 2011). Despite our findings of more frequent comorbidity in these patients, we expect that a considerable part of them may even have more unrecognized/unrecorded comorbidity. Comorbidity related to lifestyle (e.g., chronic pulmonary diseases, diabetes, and cancer) is preventable, and early intervention may slow disease progression. Hence, we see a considerable potential in increasing the focus on identifying and treating somatic comorbidity in populations with gambling disorder. It should be recognized, that after reaching these individuals and initiating the treatment of gambling disorder, joint efforts across health sectors and specialties may be needed to treat their comorbidity.

### Mortality

We observed an apparently lowered estimated adjusted mortality rate in individuals with gambling disorder, and this may be explained by either large uncertainty in the estimates or overadjustment due to more completely recorded comorbidity in individuals with gambling disorder, or a combination thereof. We cannot rule out that the mortality is lowered in the period following help-seeking and following increased care, but it does not seem likely as they have increased comorbidity, criminality, and unhealthy lifestyle.

### Components in Costs Analyses

We included costs of a variety of both welfare, income, and primary and secondary healthcare services, but we did not have data on municipally-covered rehabilitation, companions for disabled, and transport related to healthcare services. Additionally, we were not able to estimate the direct costs related to the increased criminality associated with gambling disorder, but we expect these costs to contribute marginally to the total costs as recently shown in Norway (Kristensen et al., 2022). Furthermore, our cost estimates did not include lost productivity in relatives or intangible costs, e.g., lost quality of life. Previous studies from Norway and Sweden included relevant components that we did not, including treatment of gambling disorder outside publicly funded centers, debt counseling and management, preventive measures, research, and authority efforts against gambling-related harm (Hofmarcher et al., 2020; Kristensen et al., 2022). Yet, the cost estimates in these studies were partly based on extrapolations of estimates from other countries, and this may distort the estimated costs due to differences in costs across countries, settings, and data sources (Hofmarcher et al., 2020; Kristensen et al., 2022). Hence, our cost estimates are not directly comparable to those from these previous studies (Hofmarcher et al., 2020; Kristensen et al., 2022).

### Transportability

The incomplete data coverage likely affected the estimates of occurrence and total costs, but not the estimates of comorbidity, criminality, and costs per person with gambling disorder. Of note, we focused on patients with clinically verified gambling disorder, and the total costs of problem gambling in general are expectedly much higher, as reported in studies based on population surveys (Browne et al., 2017; Hofmarcher et al., 2020; Annalisa Belloni, 2021; Kristensen et al., 2022).

Moreover, our findings of comorbidity, criminality, and costs per person may not be fully transportable to individuals with undiagnosed/untreated gambling disorder. Yet, we do not know whether these individuals have higher or lower costs, comorbidity, and criminality than those with diagnosed/treated gambling disorder. On the one hand, individuals seeking treatment may be the subgroup with more severe gambling disorder, as they more urgently need help. On the other hand, seeking treatment requires mental resources, and it is not unlikely that the individuals not seeking help are the ones with least resources, more comorbidity, more criminality, and more severe gambling issues. We found three-fold lower annual costs per individual with gambling disorder (€2934) than the reported yearly direct costs per individual with problem gambling in Norway (approximately €9000) (Kristensen et al., 2022). This may be due to our focus on healthcare and welfare services, whereas the Norwegian studies included more costs elements, as mentioned above.

### Costs Analysis Methods

Of note, we used the human capital approach to estimate costs of gambling disorder, and this method may overestimate the costs in some settings. However, as gambling disorder is a chronic disorder without an acute onset, we could not apply the friction cost method to avoid this (Drummond, 2005). Furthermore, decomposing the costs of specific disorders (e.g., gambling disorder) when complexly related to comorbidity (e.g., other mental disorders) is impossible, and it would lead to underestimation of the costs of illness of the disorder (Sortso et al., 2016; Sundqvist & Rosendahl, 2019). Additionally, for indirect costs analyses, we compared the patients to randomly selected individuals from the general population matched by sex and age only, and not by other factors potentially related to personal income. Consequently, our cost estimates do not only reflect the costs caused by gambling disorder, but rather the total use of healthcare services (direct costs) and the total difference in income between individuals with gambling disorder compared to the general population (indirect costs).

Finally, recent surveys have reported changes in the occurrence and demographics of individuals with gambling disorder over the past few years. This may affect the occurrence, comorbidity, criminality, and costs of individuals with gambling disorder both currently and in the years to come (Annalisa Belloni, 2021; Rambøll, 2022).

## Conclusion

Although we sampled the largest gambling disorder cohort in Denmark to date by supplementing the available population-based registry data with data collected from specialized treatment centers, we were restricted from drawing a firm conclusion on the occurrence and total costs of gambling disorder in Denmark. However, the high-quality individual-level data revealed a high burden of mental comorbidity in individuals with gambling disorder. Moreover, individuals with gambling disorder commonly had somatic comorbidity, e.g., chronic pulmonary disease, and they had an increased use of prescribed medication and sentenced criminality compared with the general population. Additionally, individuals with gambling disorder had considerable attributable direct and indirect costs of illness. The reasons for and consequences of these negative features associated with gambling disorder should be examined. With the increasing burden of gambling disorder, there is an urgent need for establishing a research infrastructure to address the associated health and criminality issues on both individual and societal level.

### Supplementary Information

Below is the link to the electronic supplementary material.

Supplementary file1 (DOCX 37 kb)

## Data Availability

Aggregated data are available as presented in the paper and in the supplementary material. According to Danish legislation, our approvals for using the Danish data sources in the present study do not allow us to share individual-level data with other parties. Information about access to Danish registry data is accessible through Statistics Denmark (website: https://www.dst.dk/en/TilSalg/Forskningsservice, e-mail: dst@dst.dk).
