# Supplementary material for: Comorbidity, Criminality, and Costs of Patients Treated for Gambling Disorder in Denmark
Source: J Gambl Stud. 2023 Oct 9;39(4):1765–80. doi: 10.1007/s10899-023-10255-6 (PMC10627974; doi:10.1007/s10899-023-10255-6)
Supplement: Supplementary file 1 — Supplementary file1 (DOCX 37 kb) [file 10899_2023_10255_MOESM1_ESM.docx]

# Appendix

## Codebooks

### Charlson Comorbidity Index (CCI)

| **Disease** | **ICD-8** | **ICD-10** | **Score** |
| --- | --- | --- | --- |
| Myocardial infarction | 410 | I21;I22;I23 | 1 |
| Congestive heart failure | 427.09; 427.10; 427.11; 427.19; 428.99; 782.49 | I50; I11.0; I13.0; I13.2 | 1 |
| Peripheral vascular disease | 440; 441; 442; 443; 444; 445 | I70; I71; I72; I73; I74; I77 | 1 |
| Cerebrovascular disease | 430-438 | I60-I69; G45; G46 | 1 |
| Dementia | 290.09-290.19; 293.09 | F00-F03; F05.1; G30 | 1 |
| Chronic pulmonary disease | 490-493; 515-518 | J40-J47; J60-J67; J68.4; J70.1;  J70.3; J84.1; J92.0; J96.1; J98.2; J98.3 | 1 |
| Connective tissue disease | 712; 716; 734; 446; 135.99 | M05; M06; M08; M09;M30;M31;  M32; M33; M34; M35; M36; D86 | 1 |
| Peptic ulcer disease | 530.91; 530.98; 531-534 | K22.1; K25-K28 | 1 |
| Mild liver disease | 571; 573.01; 573.04 | B18; K70.0-K70.3; K70.9; K71; K73; K74; K76.0 | 1 |
| Diabetes (type 1 or type 2) | 249.00; 249.06; 249.07; 249.09; 250.00; 250.06; 250.07; 250.09 | E10.0, E10.1; E10.9; E11.0; E11.1; E11.9 | 1 |
| Hemiplegia | 344 | G81; G82 | 2 |
| Moderate to severe renal disease | 403; 404; 580-583; 584; 590.09; 593.19; 753.10-753.19; 792 | I12; I13; N00-N05; N07; N11; N14; N17-N19; Q61 | 2 |
| Diabetes with end organ damage (type 1 or type 2) | 249.01-249.05; 249.08; 250.01-250.05; 250.08 | E10.2-E10.8; E11.2-E11.8 | 2 |
| Any tumor | 140-194 | C00-C75 | 2 |
| Leukemia | 204-207 | C91-C95 | 2 |
| Lymphoma | 200-203; 275.59 | C81-C85; C88; C90; C96 | 2 |
| Moderate to severe liver disease | 070.00; 070.02; 070.04; 070.06; 070.08; 573.00; 456.00-456.09 | B15.0; B16.0; B16.2; B19.0; K70.4; K72; K76.6; I85 | 3 |
| Metastatic solid tumor | 195-198; 199 | C76-C80 | 6 |
| AIDS | 079.83 | B21-B24 | 6 |

### Neurologic or psychiatric disorders

|  | *ICD-10* | | *ATC* | | *Indication codes* | |
| --- | --- | --- | --- | --- | --- | --- |
|  | *Included codes* | *Excluded subcodes* | *Included codes* | *Excluded subcodes* | *Included codes* | *Excluded subcodes* |
| Alcohol abuse | F10 E244 G312 G621 G721 I426 K292 K70 K852 K860 Q860 | F100 | V03AA N07BB |  |  |  |
| Anxiety disorders | F40 F41 F42 |  | N06A |  | 371 830 163 |  |
| Bipolar disorder | F30 F31 |  | N05AN01 |  |  |  |
| Brain tumors | C70 C71 C72 D32 D33 D42 D43 |  |  |  |  |  |
| Cerebral palsy | G80 |  |  |  |  |  |
| Dementia | F00 F01 F02 F03 G30 G310B G311 G318 G319 |  | N06D |  | 330 331 329 838 |  |
| Depression | F32 F33 |  | N06A |  | 168 270 |  |
| Developmental and behavioural disorders | F84 F9 | F99 |  |  |  |  |
| Drug abuse | F1 | F10 F17 | N07BC01 N07BC51 |  |  |  |
| Eating disorders | F500 F501 F502 F503 |  |  |  |  |  |
| Epilepsy | G40 |  |  |  |  |  |
| Headache | G43 G44 |  | N02C |  | 56 269 153 |  |
| Infections of the CNS | G0 A17 A321 A327 A390 A521 A522 A523 A692 A83 A84 A85 A87 A89 B003 B004 B010 B011 B020 B021 B582 B451 B375 | G08 G09 |  |  |  |  |
| Intellectual disability | F7 Q90 Q992 |  |  |  |  |  |
| Multiple sclerosis | G35 |  |  |  |  |  |
| Neuromuscular disorders | G70 G71 G72 G73 |  |  |  |  |  |
| Other neurodegenerative disorders | G10 G11 G122G G13 G14 | G130 G139 |  |  |  |  |
| Parkinson’s disease | G20 G23 |  | N04BA N04BB N04BD N04BX |  |  |  |
| Personality disorders | F60 |  |  |  |  |  |
| Polyneuropathy | G60 G61 G62 G63 G64 G130 |  |  |  |  |  |
| Schizophrenia spectrum disorders | F2 |  |  |  |  |  |
| Sleep disorders | F51 G470 G471 G473 G474 |  | N05CF01 N05CH01 |  | 372 165 166 373 170 |  |
| Stress related disorders | F43 |  |  |  |  |  |
| Stroke | I60 I61 I63 I64 | I608 |  |  |  |  |
| Traumatic brain injury | S020 S021 S027 S029 S06 | S060 |  |  |  |  |

### Medical drugs – ATC-codes

| *Substance group* | *ATC group* |
| --- | --- |
| Alimentary tract and metabolism | A |
| Blood and blood forming organs | B |
| Cardiovascular system | C |
| Dermatologicals | D |
| Genito urinary system and sex hormones | G |
| Systemic hormonal preparations, excl. Sex hormones and insulins | H |
| Anti-infectives for systemic use | J |
| Antineoplastic and immunomodulating agents | L |
| Musculo-skeletal system | M |
| Nervous system | N |
| Antiparasitic products, insecticides and repellents | P |
| Respiratory system | R |
| Sensory organs | S |
| Various | V |

### Criminality

| Group | Variable | Codes |
| --- | --- | --- |
| Type of sentence | Unsuspended/partly suspended | 111 -118 |
|  | Suspended | 121-124 |
|  | Fines, disqualification of driving license, etc. | 211; 215-224 |
| Type of crime | Shoplifting | 1332 |
|  | Burglary | 1316; 1320 |
|  | Fraud | 1357; 1360 |
|  | Forgery | 1304; 1308 |
|  | Common assault | 1252 |

## Supplementary Tables

**Supplementary Table 1: Occurrence of newly registered gambling disorder (incidence) during 2013-2017, and any registered gambling disorder (prevalence) per 1 January 2018 in Denmark.**

|  | **No. of prevalent individuals, 2018** | **Population prevalence, % (95% CI)** | **No. of incident individuals, 2017** | **Incidence rate per 100,000 person-year (95% CI)** |
| --- | --- | --- | --- | --- |
| Gambling disorder | 1,381 | 0.02 (0.02-0.03) | 532 | 9.20 (8.45-10.01) |
